# Association Between COVID-19 Exposure Duration on Receptive and Expressive Language Development in Preschool Children

**DOI:** 10.3390/children12121637

**Published:** 2025-12-01

**Authors:** Christine Sol Lee, Sangwon Hwang

**Affiliations:** Department of Rehabilitation Medicine, Daejeon Eulji University Hospital, Eulji University, Daejeon 35233, Republic of Korea; 20251154@eulji.ac.kr

**Keywords:** COVID-19, language development, children, pandemic impact, language disorders

## Abstract

**Highlights:**

**What are the main findings?**

- Longer exposure to the COVID-19 pandemic was associated with delayed receptive and expressive language development.
- Preschool-aged children are most vulnerable to prolonged pandemic effects.

**What are the implications of the main findings?**

- Children who experienced COVID-19 during critical developmental periods are at higher risk of language delays.
- Early monitoring and intervention are important to mitigate delays associated with extended pandemic exposure.

**Abstract:**

**Background/Objectives**: The COVID-19 pandemic substantially altered children’s daily experiences, limiting social interactions, which are critical for language development. This study aims to explore how the pandemic influenced receptive and expressive language abilities in children under six years, focusing on the impact of exposure duration and differences with the pandemic period. **Methods**: This retrospective cross-sectional study reviewed 189 children assessed for language delay at our outpatient clinic between 2018 and 2021. Only children evaluated using the Sequenced Language Scale for Infants or the Preschool Receptive-Expressive Language Scale were included. Participants were categorized by assessment period: pre-COVID (2018–2019), acute-COVID (2020), and chronic-COVID (2021), and by age (toddlers vs. preschoolers). Linear regression assessed the relationship between pandemic exposure duration and language scores; non-parametric tests compared groups. **Results**: During the acute-COVID period, longer exposure was associated with lower receptive and expressive percentile scores. In preschoolers, receptive scores were significantly lower in the chronic-COVID group than in the pre-COVID group, while expressive scores were lower in the chronic-COVID group than in the acute-COVID group. **Conclusions**: Prolonged exposure to the pandemic environment was associated with measurable delays in both receptive and expressive language development, especially in preschoolers. The results suggest that pandemic restrictions can hinder children’s language acquisition, indicating the need to strengthen early screening and provide timely interventions to support their developmental recovery.

## 1. Introduction

In December 2019, the detection of severe acute respiratory syndrome coronavirus 2 initiated the COVID-19 pandemic, which quickly evolved into a global public health crisis with widespread physical, psychological, and social repercussions. Countries implemented various regulations to control virus spread [1]. In South Korea, after large-scale outbreaks in 2020, the government announced mask mandates, school closures, and social distancing measures. As the virus continued to persist, South Korea entered a phase of chronic pandemic management in 2021 [1].

Beyond its physiological effects, COVID-19 profoundly affected children’s development, particularly in language acquisition, social interaction, and psychological health, largely due to mask mandates and prolonged social distancing.

Receptive and expressive language development begins immediately after birth. From smiling and crying, children gradually acquire the ability to follow commands, say first words, and understand sentences. By age three, toddlers can typically follow three-step commands, use two-word or longer phrases, and begin to use complex grammar, such as forming questions. Preschool-aged children develop the capacity to understand concepts, humor, and metaphors, while also expressing more sophisticated grammar, telling stories, and offering explanations [2]. These milestones must be achieved at the appropriate ages because children have a critical period during which sufficient linguistic input is essential for successful language acquisition [3].

Failure to achieve these developmental milestones may indicate developmental language disorder (DLD), a neurodevelopmental disorder characterized by deficits in receptive and/or expressive language in the absence of other major impairments [4,5,6]. The COVID-19 pandemic disrupted the conditions necessary for language development. Quarantine, prolonged social isolation, and limited peer interaction heightened psychosocial stress and reduced opportunities for various communicative input, all of which may negatively affect receptive and expressive abilities [7,8,9,10]. Indeed, a recent study demonstrated that infants born during the COVID-19 pandemic exhibited significantly lower cognitive performance than pre-pandemic cohorts [11].

Given the critical nature of early language development and the widespread disruption of environmental inputs during the pandemic, a significant knowledge gap remains regarding how the duration of pandemic exposure specifically impacts children’s language outcomes. This study is designed to address this gap by investigating the association between length of pandemic exposure and language outcomes in children under six years, and to identify vulnerable cohorts requiring timely intervention. We hypothesize that prolonged exposure to pandemic-related restrictions negatively affects language development. In addition, recognizing the unique impact of intellectual disabilities (IDs) on language development, we conducted a sub-cohort analysis excluding children with IDs to more clearly evaluate the effects of pandemic exposure itself.

## 2. Materials and Methods

### 2.1. Participants

**Figure 1:**
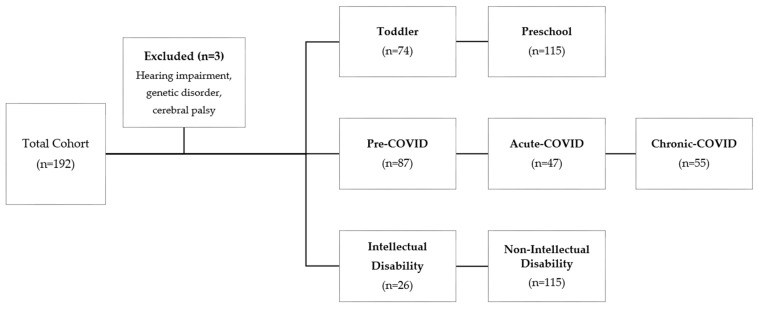
STROBE flowchart of study cohort.

We retrospectively analyzed the medical records of children aged 6 years or younger who were assessed for language disorders between January 2018 and December 2021 in the Department of Rehabilitation at Eulji Medical Center, Daejeon (Figure 1). Inclusion criteria covered those evaluated with either the Sequenced Language Scale for Infants (SELSIs) or the Preschool Receptive-Expressive Language Scale (PRES). The exclusion criteria consisted of hearing impairment, genetic or chromosomal disorders, and cerebral palsy. Children with intellectual disabilities (IDs) were analyzed separately as a sub-cohort. Given that all participants were referred for suspected language delay, the generalizability of findings may be restricted to similar clinical populations.

The study cohort was categorized into three groups according to the timing of language assessments relative to the COVID-19 pandemic: the pre-COVID group (2018–2019), the acute-COVID group (2020), and the chronic-COVID group (2021) [12]. Participants were also categorized by age: the toddler group included children up to 3 years of age (≤36 months), and the preschool group included children aged 3–6 years (36–72 months). This classification is based on internationally accepted standards, including guidelines from the American Academy of Pediatrics. The participants’ characteristics are presented in Table 1. The study protocol was reviewed and approved by the hospital’s Institutional Review Board (EMC 2023-05-005). Given the retrospective nature of the research, the requirement for obtaining informed consent was waived.

### 2.2. Language Ability Assessment and Procedure

Language ability was assessed using the SELSI or PRES, both validated screening tools for developmental language delay in Korea. The SELSI and PRES are widely used instruments that examine syntax, semantics, and pragmatics. The SELSI comprises 112 questions, with 56 assessing receptive language and 56 assessing expressive language. Similarly, the PRES comprises 90 questions, with 45 assessing receptive language and 45 assessing expressive language [2].

Raw scores were calculated separately for receptive and expressive domains and compared with normative data from age-matched peers to obtain percentile ranks. Scores were also converted into language-equivalent ages, which were then compared with chronological ages to calculate the developmental quotient (DQ). Percentile ranks and standard scores were used to determine each child’s relative standing within the population and to interpret the severity of the language disorder.

Assessments were conducted by a speech-language therapist using either the SELSI or PRES. Both scales have demonstrated validity within the Korean population [13,14]. All assessments during the study period were performed by a single experienced therapist employed at our institution since 2017, ensuring consistency in assessment procedures and minimizing risks of evaluator bias.

Children under 3 years of age were assessed using the SELSI, while those aged 3 years and above were evaluated with the PRES. In cases where both tools were administered, age-appropriate results were used for analysis.

SELSI assessments primarily relied on caregiver interviews and observational methods. In contrast, PRES assessments required direct interaction between the examiner and the child. For children over 3 years of age who were unsuitable for PRES due to limited cooperation, the SELSI was administered instead, and age-matched percentile scores were determined accordingly. We analyzed the association between SELSI and PRES percentile scores and the duration of COVID-19 exposure. The duration of exposure was calculated as the interval from 20 January 2020, the date of the first confirmed COVID-19 case in South Korea, to the child’s assessment date. This duration was converted to months by dividing the number of days by 30 and was treated as a continuous variable. Articulation and phonological accuracy were assessed using the Urimal Test of Articulation and Phonology (U-TAP), a standardized tool widely used in Korean clinical practice.

Among children with language delay, those aged 3 years or older with receptive language delay were additionally evaluated for cognitive function using the Korean Wechsler Preschool and Primary Scale of Intelligence–IV. ID was diagnosed when the full-scale intelligence quotient was below 70.

### 2.3. Statistical Analyses

Percentile scores from the receptive and expressive domains of both the SELSI and PRES were used in the analyses. To compare demographic variables among the ID and non-ID groups, the chi-square test was employed. Linear regression analysis was performed to analyze the relationship between SELSI/PRES percentile scores and the duration of pandemic exposure in children assessed between 20 January 2020 (the date of the first confirmed COVID-19 case in the Republic of Korea) and 19 January 2021. Linear regression was applied only to children assessed during the acute-COVID period because exposure duration could not be uniformly defined during the chronic phase. Multiple regression models, including age, sex, ID, and assessment tool type, were initially explored as potential covariates. However, because formal ID assessment is rarely conducted in children under 5–6 years of age, ID status was available only for a small subset of participants. After listwise deletion, fewer than ten complete cases remained, resulting in unstable coefficients with inflated standard errors and wide confidence intervals. Therefore, multivariable models were deemed statistically inappropriate and were not used in the primary analyses. Explanations are provided in the Supplementary Material for transparency.

In the subsequent chronic-COVID period (2021), the exposure timeline became inherently overlapping and heterogeneous because of varying policy changes and adaptation effects, making regression analysis on duration less interpretable. Therefore, group comparisons were employed across the pre-, acute-, and chronic-COVID periods. For comparison between different groups, the Shapiro–Wilk test was performed to validate normality, and non-parametric tests were selected because of the non-normal distribution of language percentile scores. The Kruskal–Wallis test was used to compare SELSI/PRES and U-TAP scores among groups, and post hoc Mann–Whitney U tests were performed to analyze differences across the pre-, acute-, and chronic-COVID groups. The Bonferroni correction was utilized to control for type I error inflation due to multiple comparisons, adjusting the significance level accordingly. A *p*-value of <0.05 was considered statistically significant.

All statistical analyses were conducted using the Statistical Package for the Social Sciences for Windows (SPSS, version 27.0; IBM, Armonk, NY, USA).

## 3. Results

### 3.1. Participants’ Characteristics

A total of 189 patients were reviewed, comprising 133 boys and 56 girls. Table 1 provides an overview of patient characteristics. Among them, 74 were younger than three years, and 115 were between three and six years of age. A total of 122 participants were born full term. At the time of assessment, 70 children were only children, whereas 119 had siblings. Fourteen children had a history of lingual frenectomy, and forty-five had previously received speech therapy. Eighty-four patients were assessed with SELSI, and one hundred and five with PRES. Participants were assessed across three periods: pre-COVID (2019, *n* = 87), acute-COVID (2020, *n* = 47), and chronic-COVID (2021, *n* = 55). Cognitive function was assessed in 32 patients, and those with a full-scale IQ below 70 were classified as having an intellectual disability. Participant characteristics for each COVID period group are summarized in Table 2. Table 3 presents baseline characteristics of patients with and without intellectual disability. No significant differences were detected among these groups with respect to sex, age, birth history, delivery method, presence of siblings, history of lingual frenectomy, history of speech therapy, or language assessment tools, supporting the comparability of subsequent analyses.

### 3.2. Association Between COVID-19 Exposure Duration and Language Ability Levels

**Figure 2:**
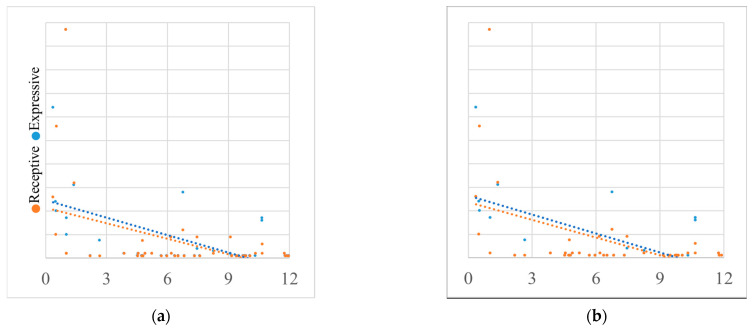
Linear regression graph showing the relationship between SELSI/PRES percentile scores and pandemic exposure in 2020. (**a**) Total cohort; (**b**) sub-cohort excluding patients with intellectual disability. Abbreviations: COVID, coronavirus disease 2019; SELSI, Sequenced Language Scale for Infants; PRES, Preschool Receptive-Expressive Language Scale.

During the first year following the initial confirmed COVID-19 case in South Korea (January 2020–January 2021), longer exposure to the pandemic was significantly associated with poorer receptive and expressive language abilities in children (*n* = 47), as shown in Figure 2. Table 3 shows linear regression analysis where exposure duration explained 24.2% of the variance in receptive language percentile scores (standardized coefficient = −0.499, *p* = 0.0004) and 21.1% of the variance in expressive language percentile scores (standardized coefficient = −0.461, *p* = 0.001). Thus, longer exposure duration was consistently associated with reduced receptive and expressive outcomes. Table 4 and Table 5 show consistent results when the same analysis was applied to the total cohort and sub-cohorts of patients without ID.

However, when the analysis was expanded to include all children assessed from the beginning of the pandemic through 2021, no significant association was observed between exposure duration and language percentile scores.

**Table 4:** Summary of linear regression analysis of SELSI/PRES percentile scores in relation to pandemic exposure in 2020 (acute-COVID period).

	Unstandardized Coefficient (B)	SE	Standardized Regression Coefficient (β)	R^2^	95% CI	*f*2	*p*-Value
Receptive	−2.48	0.65	−0.49	0.24	[−3.80, −1.16]	0.32 (medium)	0.0004
Expressive	−2.19	0.63	−0.46	0.21	[−3.47, −0.92]	0.27 (medium)	0.0011

Abbreviations: SE, standard error.

**Table 5:** Summary of linear regression analysis of SELSI/PRES percentile scores in relation to pandemic exposure in 2020 within sub-cohorts of patients excluded for IDs.

	Unstandardized Coefficient (B)	SE	Standardized Regression Coefficient (β)	R^2^	95% CI	*f*2	*p*-Value
Receptive	−2.55	0.72	−0.49	0.24	[−4.00, −1.11]	0.31 (medium)	0.0009
Expressive	−2.31	0.69	−0.46	0.21	[−3.70, −0.92]	0.27 (medium)	0.0017

Abbreviations: SE, standard error.

### 3.3. Comparison of Pre-, Acute-, and Chronic-COVID Group

**Figure 3:**
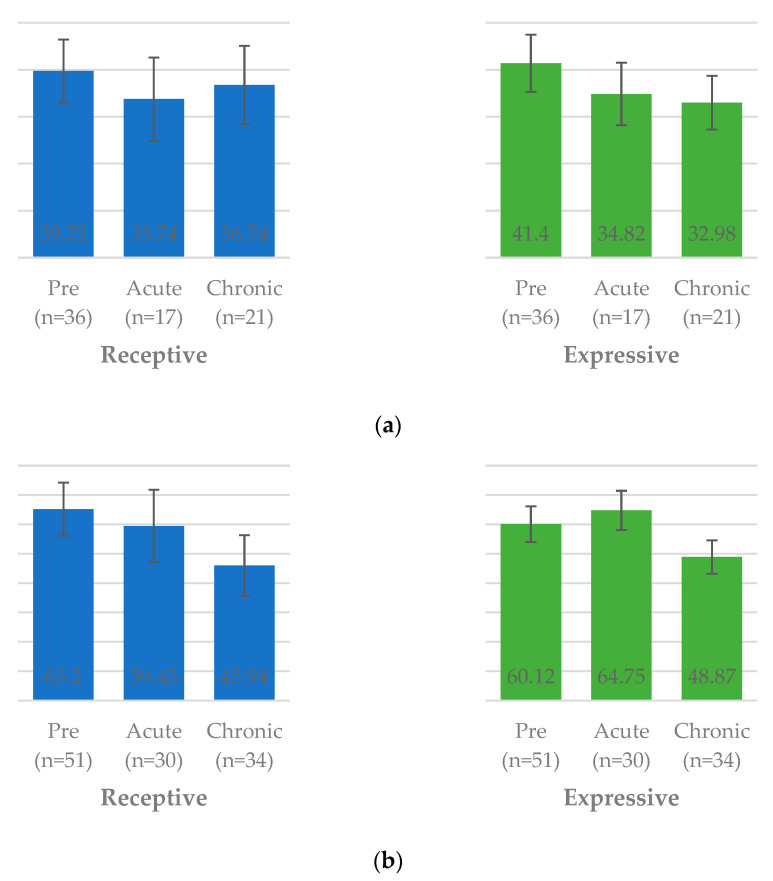
Differences in receptive and expressive language development during various periods of the COVID-19 pandemic in toddlers and preschool children. (**a**) Toddler; (**b**) preschool; abbreviations: COVID, coronavirus disease 2019.

In the preschool-aged group, receptive language percentile scores differed significantly across the pre-COVID, acute-COVID, and chronic-COVID periods. Respective average ranks were 65.2 (95% CI 56.15, 74.24), 59.43 (95% CI 47.09, 71.77), and 45.94 (95% CI 35.58, 56.3), as shown in Figure 3. Post hoc analysis indicated that children assessed during the chronic-COVID period had significantly lower receptive language percentile scores compared to those assessed in the pre-COVID period (*p* = 0.007).

For expressive language in the preschool group, the average ranks were 60.12 (95% CI 50.62, 69.61), 64.75 (95% CI 53.13,76.37), and 48.87 (95% CI 48.87, 38.48) in the pre-, acute-, and chronic-COVID groups, respectively. Although no statistically significant difference was found among the three groups overall (*p* = 0.119), post hoc analysis revealed that expressive percentile scores were significantly lower in the chronic-COVID group compared to the acute-COVID group (*p* = 0.040).

In the toddler group, no significant differences were observed in receptive or expressive language percentile scores across the three periods. However, a downward trend was observed in the chronic-COVID group, particularly in the receptive domain.

Among patients who were also assessed for U-TAP, no significant differences were found in word-unit consonant accuracy (*p* = 0.785), word-unit vowel accuracy (*p* = 0.773), sentence-unit consonant accuracy (*p* = 0.698), or sentence-unit vowel accuracy (*p* = 0.826). These findings suggest that articulation and phonological accuracy were not significantly influenced by the pandemic.

## 4. Discussion

This study indicates that prolonged exposure to the pandemic is associated with greater developmental language delays, particularly in preschool-aged children. To our knowledge, this is the first research to show an association between the duration of pandemic exposure and both receptive and expressive language outcomes.

The negative effects were most evident during the acute phase of 2020 when strict mask-wearing and social distancing limited face-to-face interaction and reduced access to visual speech cues. Our results align with prior evidence suggesting that reduced social interaction, limited visual speech cues due to mask-wearing, and heightened stress contribute to language delay in young children [15].

The observed associations may be explained by restrained access to natural linguistic input and diminished opportunities for reciprocal communication during the pandemic. Mask use likely impeded the perception of articulatory movements, while social distancing decreased peer and teacher interactions that are critical for expressive and receptive language growth.

Developmental language delays were found to be most pronounced in the early phase of the pandemic. We hypothesize that this attenuation reflects an adaptation effect. While language development may have been most disrupted during the acute phase, particularly due to mask use and social distancing, families and communities appear to have adapted to prolonged restrictions. Adaptation measures, such as increased parental involvement, use of transparent masks, and gradual easing of social distancing policies, may have mitigated early disruptions to language development.

Preschool-aged children were particularly affected when comparing language development across the three pandemic periods. Chronic-COVID group showed significantly lower percentile scores in both receptive and expressive languages. While we refer to ‘chronic-COVID’ to describe prolonged symptom presence, our study design is observational, and causal inferences cannot be drawn. Longitudinal studies are necessary to establish temporality and causality. In contrast, no significant differences were found among toddlers across different periods, suggesting that older children—more reliant on structured social environments and peer interactions for language development—were more vulnerable to prolonged pandemic restrictions. Toddlers, who primarily acquire language in the home environment, may have been comparatively less affected.

These findings corroborate earlier studies highlighting the adverse effects of the COVID-19 pandemic on children’s development. For instance, Shuffrey et al. (2022) reported that infants born throughout the pandemic had substantially lower scores in gross motor, fine motor, and social skills [16], while Deoni et al. (2022) observed diminished verbal, nonverbal, and cognitive performance skills relative to children born prior to the pandemic [11].

Several factors likely contributed to these outcomes. First, environmental constraints, such as mask-wearing and social distancing, limited access to facial cues and peer interaction, critical for receptive and pragmatic language development [17,18]. South Korea exhibited a remarkably high mask-wearing compliance rate of about 94%, far exceeding that of many other countries [19]. This cultural and policy-driven adherence may have amplified the reduction in facial cue exposure, suggesting that the observed impact on language development is particularly pronounced in this setting. Second, elevated stress and behavioral changes have been reported in children, including increased oppositional-defiant behaviors [20], while maternal stress has been linked to delayed language milestones [21]. These stress-related factors may reduce opportunities for verbal expression, contributing to expressive delays. Third, increased screen time emerged as another important factor during the pandemic. School closures and reduced access to out-of-home care led to 79% of families losing daycare or similar support, accompanied by a substantial increase in the frequency of screen-mediated reading [22]. Both educational and recreational screen times increased during this period. Hedderson et al. reported that, compared to the pre-pandemic period, children’s total screen time increased by 1.75 h per day, while recreational screen time increased by 0.8 h per day [23]. The literature consistently shows that increased media exposure—including that of parents—is linked to delayed language development, with higher screen time associated with poorer language outcomes in children [24,25,26]. Collectively, these factors highlight how the pandemic disrupted both the quality and quantity of linguistic input.

Despite the negative effects of the pandemic in social contexts, post-pandemic recoveries were attempted in families. Social restrictions led to increased time in the home environment. Schmeer et al. reported increased parental–child interaction, such as playing and helping with schoolwork, especially in language arts [27]. Household learning resources such as books and toys were found to be increased in chronic-COVID period compared to pre-COVID period, compensating for difficulties of school closures and learning opportunities [28].

This study has several limitations. First, the classification of the acute and chronic phases was constrained by overlapping changes in public health restrictions, which may have introduced bias. Moreover, our single-center outpatient sample may lack representativeness for the general population, potentially limiting the generalizability of findings. Environmental factors such as parental involvement, home literacy, screen time, socioeconomic status, parental interaction time, and daycare attendance were unavailable, thus restricting our ability to control for these influences. Additionally, the retrospective design inherently restricts causal inferences and may introduce unmeasured confounding variables. Also, the limited sample size, particularly within the toddler group, reduced the statistical power and may have obscured meaningful differences. Finally, given the pediatric nature of the study population, developmental heterogeneity could not be fully controlled despite stratified analyses.

Clinically, our findings underscore the importance of early monitoring and targeted intervention strategies for children—especially preschoolers—who experienced prolonged pandemic exposure during critical periods of language development. Based on these results, we recommend implementing early language screening programs. Additionally, parental education to support effective language stimulation at home and timely speech interventions is recommended. These strategies may help mitigate the pandemic’s impact on language development in children.

## 5. Conclusions

Prolonged exposure during the acute-COVID-19 pandemic phase showed a significant association with receptive and expressive language delays among children under six years. Preschool-aged children were especially vulnerable, emphasizing the need for careful observation and timely intervention in children’s critical developmental stages. Future research with prospective designs, multicenter studies with larger and more representative samples, and more comprehensive data collection is warranted to address these limitations.

## Figures and Tables

**Table 1:** Demographic information of study cohort (*n* = 189).

Variable	Participant Characteristics
COVID groups, *n* (%)	
Pre-COVID	87 (46.0)
Acute-COVID	47 (24.9)
Chronic-COVID	55 (29.1)
Sex, *n* (%)	56 (29.6)
Male	133 (70.4)
Female	56 (29.6)
Age	
Toddler (≤36 months)	74 (39.2)
Preschool (36–72 months)	115 (60.8)
Birth History, *n* (%)	
Preterm birth (≤36 weeks)	35 (18.5)
Term birth (>36 weeks)	122 (64.6)
Unanswered	32 (16.9)
Delivery, *n* (%)	
NSVD	72 (38.1)
C/S	71 (37.6)
Unanswered	46 (24.0)
Family member, *n* (%)	
Only child	70 (37.0)
Sibling	119 (63.0)
Lingual frenectomy	14
Previous language therapy	45
Assessment Method	
SELSI	84 (44.4)
PRES	105 (55.6)

Values are expressed as the number of participants (percentage). Abbreviations: ID, intellectual disability; COVID, coronavirus disease 2019; NSVD, normal spontaneous vaginal delivery; C/S, cesarean section; SELSI, Sequenced Language Scale for Infants; PRES, Preschool Receptive-Expressive Language Scale.

**Table 2:** Baseline characteristics by COVID period and age groups.

Groups	*n* (%)	Mean Age in Months (SD)	Sex	
			Male, *n* (%)	Female, *n* (%)
COVID period groups				
Pre-COVID	87 (46.0%)	41.38 (13.90)	62 (71.3%)	25 (28.7%)
Acute-COVID	47 (24.9%)	43.28 (13.08)	30 (63.8%)	17 (36.2%)
Chronic-COVID	55 (29.1%)	42.45 (14.12)	41 (74.5%)	14 (25.5%)
Age groups				
Toddler (≤36 weeks)	74 (39.2%)	28.93 (4.33)	56 (75.7%)	18 (24.3%)
Preschool (36–72 months)	115 (60.8%)	50.68 (10.54)	77 (67.0%)	38 (33.0%)

**Table 3:** Baseline characteristics compared between children with and without intellectual disability.

Variable	ID	Non-ID	*p*-Value
COVID groups, *n* (%)	26	163	0.029
Pre-COVID	10 (38.5%)	77 (47.2%)	
Acute-COVID	3 (11.3%)	44 (27.0%)	
Chronic-COVID	13 (50%)	42 (25.8%)	
Sex, *n* (%)			0.925
Male	19 (73.1%)	114 (69.9%)	
Female	7 (27.0%)	49 (30.1%)	
Age			0.236
Toddler (≤36 months)	6 (23.1%)	68 (41.7%)	
Preschool (36–72 months)	20 (76.9%)	115 (70.6%)	
Birth History, *n* (%)			0.720
Preterm birth (≤36 weeks)	5 (19.2%)	34 (20.9%)	
Term birth (>36 weeks)	18 (69.2%)	101 (62.0%)	
Unanswered	3 (11.5%)	28 (17.2%)	
Delivery, *n* (%)			0.920
NSVD	9 (34.6%)	63 (38.7%)	
C/S	10 (38.5%)	60 (36.8%)	
Unanswered	7 (26.9%)	40 (24.5%)	
Family member, *n* (%)			0.540
Only child	8 (30.8%)	64 (39.3%)	
Sibling	18 (69.2%)	99 (60.7%)	
Lingual frenectomy	1	12	
Previous language therapy	2	38	
Assessment Method			0.410
SELSI	14 (53.8%)	70 (42.9%)	
PRES	12 (46.2%)	93 (57.1%)	

Values are presented as the number of participants (percentage). Chi-square test was used to compare sample sizes between groups. Abbreviations: ID, intellectual disability; COVID, coronavirus disease 2019; NSVD, normal spontaneous vaginal delivery; C/S, cesarean section; SELSI, Sequenced Language Scale for Infants; PRES, Preschool Receptive-Expressive Language Scale.

## Data Availability

The data that support the findings of this study can be obtained from the corresponding author upon reasonable request due to privacy reasons.

## Supplementary Materials

The following supporting information can be downloaded at: https://www.mdpi.com/article/10.3390/children12121637/s1, Note S1: Supplementary Note.

## Abbreviations

The abbreviations listed below are used in this manuscript:

COVID-19	Coronavirus Disease 2019
SELSI	Sequenced Language Scale for Infants
PRES	Preschool Receptive-Expressive Language Scale
U-TAP	Urimal Test of Articulation and Phonation
ID	Intellectual Disability
