# Supplementary material for: Association Between COVID-19 Exposure Duration on Receptive and Expressive Language Development in Preschool Children
Source: Children (Basel). 2025 Dec 1;12(12):1637. doi: 10.3390/children12121637 (PMC12731886; doi:10.3390/children12121637)
Supplement: Supplementary file 1 [file children-12-01637-s001.zip › children-3986414-supplementary.pdf]

Supplementary Note S1.

Multiple regression models including age, sex, ID status, and assessment tool type were initially explored. However, ID assessment was rarely available in children under 5–6 years of age, and listwise deletion resulted in fewer than ten complete cases. Therefore, no stable multivariable model could be computed, and only simple regression results are reported in the main manuscript.
